# Fractional amplitude of low-frequency fluctuations (fALFF) in post-stroke depression

**DOI:** 10.1016/j.nicl.2017.07.014

**Published:** 2017-07-18

**Authors:** Natalia Egorova, Michele Veldsman, Toby Cumming, Amy Brodtmann

**Affiliations:** a The Florey Institute of Neuroscience and Mental Health, Melbourne, Australia.; b The Nuffield Department of Clinical Neurosciences, University of Oxford, Oxford, UK.

**Keywords:** Fractional amplitude of low-frequency fluctuations (fALFF), Post-stroke depression, Stroke, Resting state functional magnetic resonance imaging (rs-fMRI), Insula, Dorsolateral prefrontal cortex (DLPFC)

## Abstract

Depression is a common outcome following stroke, associated with reduced quality of life and poorer recovery. Despite attempts to associate depression symptoms with specific lesion sites, the neural basis of post-stroke depression remains poorly understood. Resting state fMRI has provided new insights into the neural underpinnings of post-stroke depression, but has been limited to connectivity analyses exploring interregional correlations in the time-course of activity. Other aspects of resting state BOLD signal remain unexamined. Measuring the amplitude of low frequency fluctuations allows the detection of spontaneous neural activity across the whole brain. It provides complementary information about frequency-specific local neural activity.

We calculated the fractional amplitude of low frequency fluctuations (fALFF) in a group of 64 participants scanned 3 months post-stroke. Twenty showed depression symptoms when assessed with the Patient Health Questionnaire (PHQ-9). We performed analyses in both the typical 0.01–0.08 Hz range, as well as separately in the slow-5 (0.01–0.027 Hz) and slow-4 (0.027–0.073 Hz) ranges. We found significantly higher fALFF in the depressed compared to non-depressed participants in the left dorsolateral prefrontal cortex (DLPFC) and the right precentral gyrus, and a significant association between higher depression scores and higher fALFF in the left insula. The group differences were detected in the slow-5 fluctuations, while the association with depression severity was observed in the slow-4 range. We conclude that post-stroke depression can be characterised by aberrant spontaneous local neural activity, which in small samples could be a more sensitive measure than lesion volume and location.

## Highlights

• We examined the amplitude of low frequency fluctuations (ALFF) in post-stroke depression (82).
• We observed aberrant fractional ALFF in the insula and left DLPFC associated with depression (94).
• We observed no overlap between aberrant fALFF and stroke lesions (66).
• fALFF is a sensitive measure for detecting post-stroke depression (68).

## 1 Introduction

Depression is one of the most common psychiatric disorders observed in stroke patients (Loubinoux et al., 2012). The risk of occurrence is similar in early, middle and late phases of stroke recovery (Hackett et al., 2005). Post-stroke depression has been associated with cognitive impairment (Downhill and Robinson, 1994) negatively affecting stroke patients' social functioning (Mast, 2004) and quality of life (Angeleri et al., 1993, Guajardo et al., 2015). Despite many previous attempts to characterise post-stroke depression through localization and extent of stroke lesions (Grajny et al., 2016, Pujol et al., 2000, Rajashekaran et al., 2013, Shimoda and Robinson, 1999), little progress has been made. This is likely due to the complex nature of the disorder, manifesting not only in the event of a lesion to a vulnerable brain region or brain network, but also after stroke-induced functional reorganisation of the neural system altering connectivity, metabolic activity and neurovascular coupling (Carter et al., 2012, Veldsman et al., 2015).

Resting state functional magnetic resonance imaging (rs-fMRI) has been increasingly used (Carter et al., 2012, Ovadia-Caro et al., 2014) to characterise stroke beyond the effects of specific lesions. Compared to task fMRI, rs-fMRI is also more suitable for studying stroke populations due to the absence of potentially challenging tasks and assumptions underlying blood oxygen level dependent (BOLD) response (Veldsman et al., 2015). Several resting state studies in stroke participants related depression severity to reduced connectivity in the default mode network (Lassalle-Lagadec et al., 2012) and the affective network (Zhang et al., 2014). Previous resting state studies only focused on functional connectivity examining temporal correlations in BOLD signal in specific brain networks. In addition to functional connectivity one can measure the amplitude of low frequency fluctuations (ALFF) in the range of 0.01–0.08 Hz shown to reflect spontaneous neural activity of the brain (Zuo et al., 2010). In particular, the fractional amplitude of low-frequency fluctuations (fALFF) measures the relative contribution of low frequency fluctuations within a specific frequency band to the whole detectable frequency range (Zou et al., 2008). In addition to providing a way to quantify differences in resting state activity across the whole brain, fALFF reflects a different aspect of the BOLD signal. While the canonical functional connectivity quantifies temporal synchrony between distinct spatially separate regions, thereby revealing the strength of inter-regional cooperation, low frequency fluctuations allow us to study the amplitude of regional neuronal activity, potentially identifying brain areas with abnormal local functioning (Chen et al., 2015). Investigating different dimensions of resting state functioning is important, as differences may lie not only in the patterns of connectivity, but also the power of local neuronal activity.

In non-stroke populations, researchers have found both lower and higher magnitude of fALFF associated with depression (Jing et al., 2013, Lai and Wu, 2015, Wang et al., 2016). For example, Wang and colleagues showed that in depressed patients, fALFF was significantly higher in the right precentral gyrus, right inferior temporal gyrus, bilateral fusiform gyri and cerebellum; in contrast fALFF was lower in the left dorsolateral prefrontal cortex, bilateral medial orbitofrontal cortex and bilateral middle and left inferior temporal gyri, as well as the right inferior parietal lobule (Wang et al., 2012). Yet higher fALFF has been more frequently associated with depression. For instance, patients with treatment resistant depression were found to have higher fALFF in the right thalamus, right inferior frontal gyrus and inferior parietal lobule compared to non-treatment resistant depression subjects and healthy controls. Higher fALFF in the right thalamus was also shown to correlate with worse antidepressant treatment response (Yamamura et al., 2016). Similarly, recovered and currently depressed (female) patients showed higher fALFF in the right putamen compared to healthy controls; only currently depressed patients had higher fALFF in the right ventral medial frontal gyrus compared to the recovered and healthy control groups, suggesting it may be a correlate of ongoing depression (Jing et al., 2013).

While the resting state connectivity studies focus on one frequency range, typically 0.01–0.08 Hz, low frequency oscillations are typically subdivided into 4 narrower bands: slow-5 (0.01–0.027 Hz), slow-4 (0.027–0.073 Hz), slow-3 (0.073–0.198 Hz) and slow-2 (0.198–0.25 Hz) (Buzsáki and Draguhn, 2004). Slow-4 and slow-5 oscillations are most closely related to gray matter signal and most useful in identifying correlates of functional processing and disorders (Zuo et al., 2010). As different frequency bands originate from different neural sources they could relate to different aspects of brain processing. Researchers have investigated differences between slow-4 and slow-5 fluctuations in depression. For example, Wang and colleagues found an interaction between frequency (slow-4 and slow-5) and group (depression vs. healthy controls), showing that oscillations in the slow-5 range were more sensitive to MDD (Wang et al., 2016), as the difference between MDD and healthy control groups was observed in slow-5 but not slow-4 range. This finding highlights the importance of investigating not only the average 0.01–0.08 Hz range but also slow-4 and slow-5 separately.

The goal of the current study was to identify resting state correlates of post-stroke depression, focussing on local frequency-specific differences in spontaneous fluctuations. We compared depressed and non-depressed stroke participants, as well as exploring an association between the spontaneous low frequency fluctuations and the Patient Health Questionnaire (PHQ-9) (Kroenke et al., 2001) scores in all participants. PHQ-9 is brief and easy to administer, and is sensitive to depression diagnosis and grading symptom severity, specifically validated in stroke populations (Kroenke et al., 2001, Williams et al., 2005). Given the role of fALFF in depression, as well as the fact that it is less prone to noise compared to ALFF (Zou et al., 2008), we focused on the fALFF. We examined the typical 0.01–0.08 Hz range. In addition, slow-4 and slow-5 fluctuations were considered separately in order to identify potential differences in their contributions to the average signal and specialization as a post-stroke depression correlate.

## 2 Methods

### 2.1 Participants

Participants with ischemic stroke were recruited from the Stroke Units at three Melbourne hospitals: Austin Hospital, Box Hill Hospital, and the Royal Melbourne Hospital as part of the Cognition and Neocortical Volume after stroke (CANVAS) study (Brodtmann et al., 2014). Ethical approval was given by each hospital's ethics committee in line with the Declaration of Helsinki. Participants gave informed written consent; those unable to consent were excluded from participation. Ischemic stroke participants underwent cognitive and neuropsychological testing and MRI scanning at 3 months post-stroke. Participants with haemorrhagic stroke or venous infarction or significant medical comorbidities were excluded from participation. Participants were also excluded if they did not meet standard MRI safety criteria or had history of mental or psychiatric illness. The presence of psychiatric history prior to stroke was a core exclusion criterion for the entire CANVAS study.

A stroke neurologist (AB) classified strokes according to aetiology (Goldstein et al., 2001) and site of clinical presentation (Bamford et al., 2016). Impairment and disability were assessed in stroke participants using the National Institutes of Health Stroke Scale (NIHSS) (Brott et al., 1989) and Modified Rankin Scale (Rankin, 1957). All participants were interviewed for medical history, existing vascular risk factors and medications.

### 2.2 Depression assessment

Participants' depression severity was assessed using the Patient Health Questionnaire-9 (Kroenke et al., 2001). The PHQ-9 assesses depressive symptoms based on the 9 DSM-IV criteria: anhedonia, hopelessness, sleep disturbance, low energy, appetite changes, worthlessness, trouble concentrating, slowing/restlessness, self-harm. Responses to each item include 0 (‘not at all’), 1 (‘several days’), 2 (‘more than half the days’) or 3 (‘nearly every day’). Scores of 5–9 indicate mild depression, 10–14 signals moderate depression, 15–19 suggests moderately severe depression and scores above 20 to the maximum of 27 represent severe depression. Participants in the study were not medicated for depression; most had not reported their symptoms to family members or their physicians, and symptoms were only identified on screening for this project.

### 2.3 Imaging data acquisition and pre-processing

All images were acquired on a Siemens 3T Tim Trio scanner (Erlangen, Germany) with a 32 channel head coil. A high-resolution anatomical MPRAGE volume of 160 sagittal slices with 1 mm isotropic voxels, TR = 1900 ms, TE = 2.55 ms, 9° flip angle, 100% field of view in the phase direction and 256 × 256 acquisition matrix was collected. A high-resolution 3D SPACE-FLAIR image was acquired with 160 1 mm thick sagittal slices, TR = 6000 ms, TE = 380 ms, 120° flip angle, 100% field of view in the phase direction and 256 × 254 acquisition matrix. Resting state data (132 volumes taking approximately seven minutes) were acquired with axial oriented, interleaved slices, 3 mm isotropic voxels, 3 mm slice gap, TR = 3000 ms, TE = 30 ms and 85° flip angle, 100% field of view in phase direction and 72 × 72 acquisition matrix. During resting state acquisition participants were instructed to keep their eyes closed.

Functional images were pre-processed in SPM8 (Wellcome Department of Imaging Neuroscience, London, UK, http://www.fil.ion.ucl.ac.uk/spm/). Images were slice-time corrected, with the middle slice as a reference; six-parameter rigid body realignment was performed; images were co-registered to the high-resolution structural image.

Lesions were manually traced on the high-resolution FLAIR image. A stroke neurologist (AB) visually inspected and verified the manually traced images. A binary lesion mask was created used for segmentation and normalization to the MNI152 template and used with the Clinical Toolbox SPM extension (Rorden et al., 2012) to improve tissue segmentation and preserve the lesion size (Andersen et al., 2010, Ripolles et al., 2012). Tissue segmentations were manually inspected for quality assurance. Functional images were smoothed with an 8 mm full width half maximum Gaussian kernel. For lesion overlap visualisation, MRIcron software (Rorden et al., 2007) was used. Lesion volume was estimated using SPM12 (Wellcome Department of Imaging Neuroscience, London, UK, http://www.fil.ion.ucl.ac.uk/spm/).

The fALFF values were computed on detrended data using the REST software (Song et al., 2011) (State Key Laboratory of Cognitive Neuroscience and Learning in Beijing Normal University; http://resting-fmri.sourceforge.net). REST has in-built fast Fourier transform functions to convert time series data to the frequency domain and calculate the power spectrum. The ratios of the power in the 0.01–0.08 Hz frequency range, the slow-5 (0.01–0.027 Hz) and the slow-4 (0.027–0.073 Hz) range were calculated relative to the full frequency range (0–0.25 Hz). The fALFF values were then z-transformed prior to statistical analyses. All analyses were performed at the whole-brain level.

### 2.4 Statistical analysis

First, we identified participants with a PHQ-9 depression score of ≥ 5 as a cut-off for mild depression at 3 months post-stroke. We divided participants into depressed (≥ 5) and non-depressed (< 5) groups and compared the two groups on relevant characteristics using a 2-sample *t*-test (for age, PHQ-9, Boston Naming Test Z score, Digit Span Z score, Hopkins Verbal Learning Test Total Z score, Star Cancellation Test score, The Rey-Osterrieth complex figure Test Copy and Recall Z scores), Mann-Whitney-Wilcoxon test (for NIHSS at baseline) and a Chi-square test (for sex, number of participants with previous strokes, number of participants treated with tissue plasminogen activator (tPA) at baseline, number of participants with Modified Rankin Score ≥ 2 at 3 months post-stroke).

Lesion volume was compared between the groups, using a two-sample *t*-test, in addition a Pearson correlation between lesion volume and depression severity (PHQ-9 scores) was performed in all participants. Finally, we also checked whether total intracranial volume and the extent of white matter hyperintensity burden differed between the groups. The level of p < 0.05 was used to assess significance. For all imaging analyses the results were considered significant at the voxel-level p < 0.005 at the whole-brain level, cluster-corrected at p_FDR_ < 0.05.

### 2.5 fALFF in 0.01–0.08 Hz range

For the ‘depressed vs. non-depressed’ contrast fALFF maps were compared on a voxel-wise basis using a two-sample *t*-test in SPM8 with age, sex and NIHSS scores at baseline as covariates. For the assessment of the association between depression severity and fALFF, a regression with PHQ-9 values for all participants and age, sex and NIHSS scores at baseline as covariates was performed.

### 2.6 fALFF in slow-5 (0.01–0.027 Hz) and slow-4 (0.027–0.073 Hz) ranges

We repeated both the group and regression analyses for the 2 frequency ranges separately.

Our depression grouping was based on a cut-off value of PHQ-9 ≥ 5, to capture all those with depressive symptoms, even at the mild end of the scale. To assess whether there were any differences between individuals with more severe depression (PHQ-9 ≥ 10) and those with milder depressive symptoms (PHQ-9 > 5 but < 10), we extracted the values from each of the significant clusters identified in all analyses in the 0.01–0.08 Hz range and directly compared these two groups using the Mann-Whitney-Wilcoxon Test.

## 3 Results

### 3.1 Behavioural results

Sixty-four participants in the CANVAS study who had a full set of imaging data (structural and functional images) and depression scores available at 3 months post-stroke were included in the analysis. Twenty showed symptoms of mild to moderate depression (PHQ-9 score ≥ 5 at 3 months) and were assigned to the ‘depressed’ group, while others were included in the ‘non-depressed’ group. This prevalence rate of 31% is consistent with previous reports (Hackett et al., 2005, Provinciali and Coccia, 2002). The groups were not different on age or NIHSS baseline scores; there were, however, significantly more females in the depressed group compared to the non-depressed group (Table 1), which was expected based on the previous literature (Paradiso and Robinson, 1998). For the majority of participants, it was their first stroke, with no significant difference between groups on the history of stroke or level of post-stroke disability (mRS) at 3 months (Table 1). No participant had stroke recurrence at 3 months and at 12 months followup. At the time of testing, all participants were community dwelling and had been living back at home; most had returned to normal activities including driving and work. The level of cognition in all participants was sufficiently high to perform standard cognitive tasks and not to interfere with evaluation of psychiatric symptoms; cognitive performance was comparable between groups (Table 1). Age, sex and NIHSS scores were included in the statistical analyses as covariates.

**Table 1:** Group comparison of demographics and clinical outcomes. M - mean; SD - standard deviation, N - number. P-values for the *t*-test (PHQ-9 at 3 months, age, total intracranial volume, white matter hyperintensity volume, Boston naming test, Digit Span test, Hopkins Verbal Learning test, Star cancellation test, Complex figure test (copy), Complex figure test (recall); for Wilcoxon test (NIHSS at baseline); for Chi-square test (sex, N with previous stroke, N with Modified Rankin Scale (mRS) ≥ 2, N with tissue plasminogen activator (tPA) treatment).

Variable	Non-depressed	Depressed	p-Value (2-tailed)
PHQ-9 at 3 months post-stroke - M (SD)	2.07 (1.4)	8.4 (3.8)	< 0.01
N (N female)	44 (11)	20 (12)	0.01
NIHSS at baseline - median (range)	2 (0 − 10)	3 (1 − 10)	0.12
Age - M (SD)	67.72 (14.5)	67.55 (10.7)	0.96
N with previous stroke history	1	8	0.16
N with mRS score ≥ 2 at 3 months post-stroke	11	9	0.12
N treated with tPA at baseline	1	4	0.57
Total intracranial volume (ml) - M (SD)	1511 (166)	1453 (149)	0.17
White matter hyperintensity volume (ml) - M (SD)	9.44 (11.21)	9.48 (14.17)	0.99
Boston Naming Test Z score - M (SD)	0.42 (0.83)	0.30 (0.74)	0.58
Digit Span Z score - M (SD)	0.06 (0.81)	− 0.35 (0.94)	0.11
Hopkins Verbal Learning Test Total Z score - M (SD)	0.02 (1.21)	0.29 (1.01)	0.35
Star Cancellation Test score - M (SD)	53.3 (1.43)	53.4 (1.04)	0.76
Complex Figure Test Copy Z score - M (SD)	0.05 (1.34)	− 0.15 (1.29)	0.32
Complex figure Test Recall Z score - M (SD)	− 0.40 (1.79)	− 0.07 (1.00)	0.78

### 3.2 Analysis of lesion volume and location

**Fig. 1:**
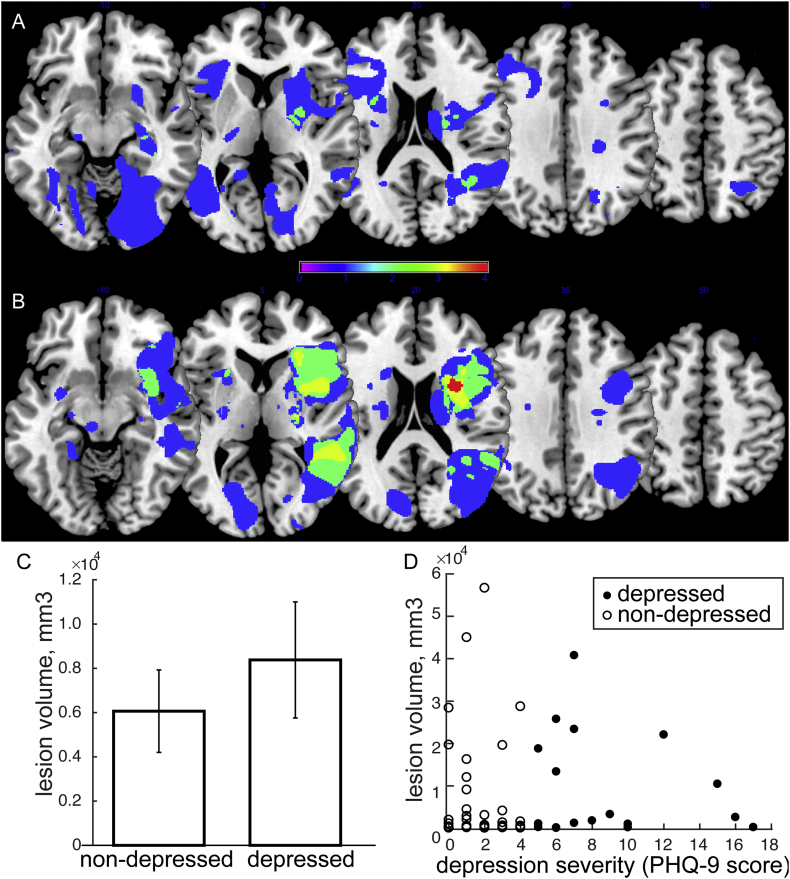
Lesion analysis. A. Lesion overlap in the depressed group. B. Lesion overlap in the non-depressed group. C. Lesion volume comparison between the groups demonstrating no significant difference between depressed and non-depressed group. D. Scatterplot demonstrating the relationship between lesion volume and depression severity and showing no significant correlation between the two variables.

Lesion overlap maps for the depressed (Fig. 1A) and non-depressed (Fig. 1B) groups failed to reveal any consistent association between the lesion location and depression level. No > 2 participants' lesions overlapped in this sample of 20. In addition, lesion volume appeared to be slightly greater in the depressed group but not significantly different between the groups (Fig. 1C), p = 0.48. A correlation between lesion volume and depression severity was also not significant (Fig. 1D) in all participants (p = 0.98), and specifically within the depressed group (p = 0.67). There were also no group difference in total intracranial volume and the extent of white matter hyperintensity (Table 1). In addition, there was no significant correlation between total intracranial volume and PHQ-9 (p = 0.37), nor was there a significant relationship between white matter hyperintensity extent and PHQ-9 (p = 0.66).

### 3.3 fALFF in 0.01–0.08 Hz range

**Fig. 2:**
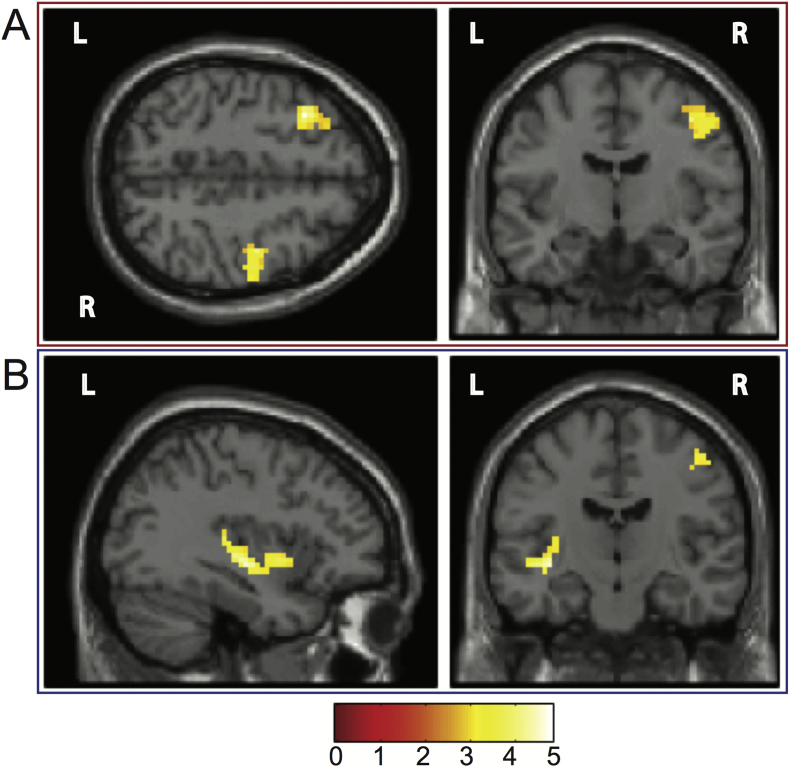
Main results in the 0.01–0.08 Hz range. A. Group comparison (depressed > non-depressed) controlling for age, sex, NIHSS. A significant cluster in the left DLPFC (− 36,21,45) and the right precentral gyrus (42,−9,48). B. A regression with PHQ-9 scores in all participants controlling for age, sex, NIHSS. A significant cluster in the left insula/superior temporal gyrus (− 36,3,−3). A cluster in the right precentral gyrus (48,− 9,54) is significant at the voxel level but does not survive cluster-correction. The colour bar represents T and F statistics for A and B respectively.(For interpretation of the references to colour in this figure legend, the reader is referred to the web version of this article.)

A direct comparison between depressed and non-depressed patients showed a significant group difference in the left dorsolateral prefrontal cortex and the right precentral gyrus (Table 2 and Fig. 2A). A regression with the PHQ-9 scores in all patients showed a significant cluster in the posterior left insula/superior temporal gyrus. A cluster (k = 25) in the right precentral gyrus similar to the one appearing in the group comparison was significant at the voxel-level (p = 0.002, T = 2.94, Z = 2.82, MNI: 48, − 9, 54) but not at the cluster-corrected level p_FDR_ = 0.59 (Table 2 and Fig. 2B).

**Table 2:** Significant results z-fALFF 0.01–0.08.

Region	Hemisphere	x	y	z	Peak p(unc)	Cluster p(FDR-corr)	k	T	Z
zfALFF 0.01–0.08 depressed > non-depressed patients (age, sex, NIHSS)									
Dorsolateral prefrontal cortex (DLPFC)	Left	− 36	21	45	< 0.001	0.046	92	4.81	4.4
		− 30	15	51	< 0.001			4.03	3.78
		− 12	27	57	< 0.001			3.46	3.29
Precentral gyrus	Right	42	− 9	48	< 0.001	0.032	116	4.04	3.78
		51	− 12	48	< 0.001			3.54	3.36
		45	− 12	39	0.001			3.46	3.29

zfALFF 0.01–0.08 regression PHQ-9 (age, sex, NIHSS)									
Insula	Left	− 36	− 15	− 6	< 0.001	0.013	135	4.07	3.8
		− 36	3	− 3	0.001			3.45	3.28
		− 36	− 27	9	0.001			3.36	3.2

### 3.4 fALFF in slow-5 (0.01–0.027 Hz) and slow-4 (0.027–0.073 Hz) ranges

A group difference between depressed and non-depressed patients in the slow-5 range revealed a significant difference in the right precentral gyrus and supplemental motor/middle frontal cortex (Table 3). No significant differences between the groups were observed in the slow-4 range, however, a cluster (k = 24) in the left DLPFC showed a trend at the voxel-level p = 0.001, T = 3.3, Z = 3.15, MNI: − 33, 21, 48 that did not survive at the cluster-corrected level p_FDR_ = 0.56.

**Table 3:** Significant results z-fALFF for slow-5 and slow-4 oscillations.

Region	Hemisphere	x	y	z	Peak p(unc)	Cluster p(FDR-corr)	k	T	Z
Slow-5 (0.01–0.027 Hz) depressed > non-depressed (age, sex, NIHSS)									
Supplementary motor cortex/middle frontal gyrus	Right	9	− 9	63	< 0.001	0.002	192	4.12	3.85
		3	− 6	45	< 0.001			3.75	3.54
		0	6	60	< 0.001			3.47	3.3
Precentral gyrus	Right	48	− 9	45	< 0.001	0.051	92	4.08	3.81
		57	− 15	36	0.002			3.1	2.97
		39	− 18	39	0.002			2.99	2.87

Slow-5 (0.01–0.027 Hz) regression PHQ-9 (age, sex, NIHSS)									
None									

Slow-4 (0.027–0.073 Hz) depressed > non-depressed (age, sex, NIHSS)									
None									

Slow-4 (0.027–0.073 Hz) regression PHQ-9 (age, sex, NIHSS)									
Insula/superior temporal gyrus	Left	− 33	− 15	6	< 0.001	0.001	195	4.84	4.42
		− 39	− 18	− 3	< 0.001			4.58	4.22
		− 39	− 27	9	< 0.001			4.52	4.17

A regression with PHQ-9 scores in all participants did not show any significant clusters in the slow-5 range. In the slow-4 range, however, a cluster in the left insula/superior temporal gyrus was significantly positively associated with the PHQ-9 scores, Table 3.

**Fig. 3:**
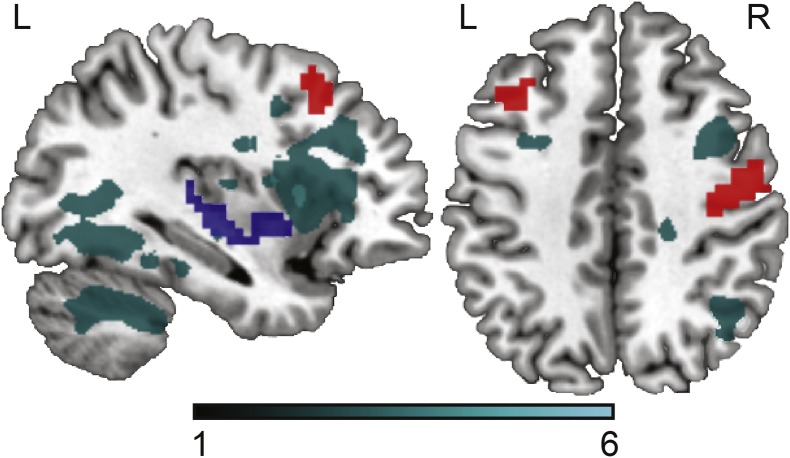
Lesion overlap map for all participants is shown in cyan; the results of the group comparison (depressed vs. non-depressed) are shown in red; the result of the regression analysis (PHQ-9 scores) is shown in blue.(For interpretation of the references to colour in this figure legend, the reader is referred to the web version of this article.)

In order to check whether the fALFF results overlapped with stroke lesions in all participants, we overlaid them in one image, shown in Fig. 3. No overlap was observed in any participant.

**Fig. 4:**
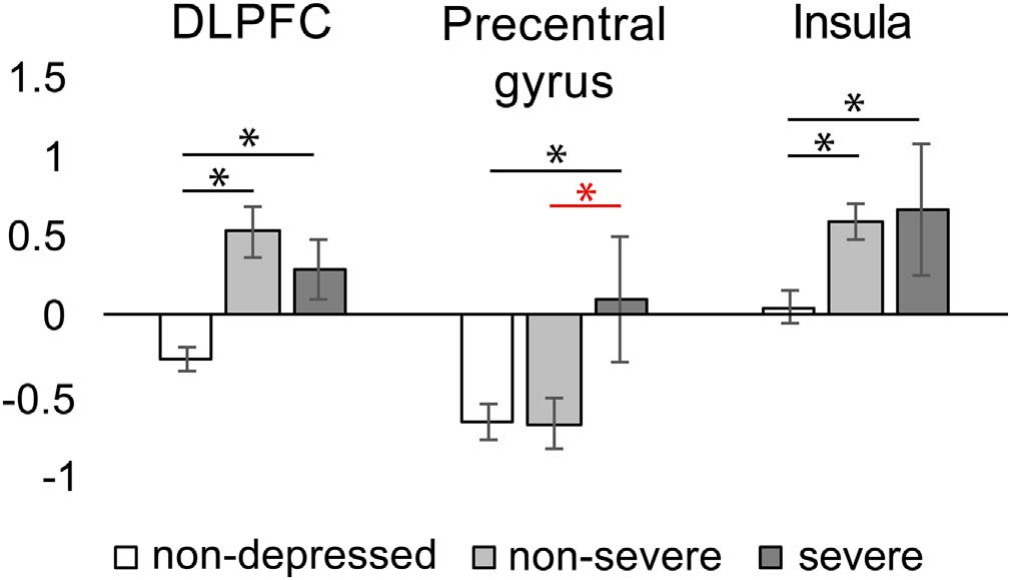
Average fALFF values extracted from significant clusters in the 0.01–0.08 Hz range analyses (DLPFC, Precentral gyrus, insula) by depression severity: non-depressed (PHQ-9 < 5), non-severe depression (PHQ-9 ≥ 5 but < 10), severe depression (PHQ-9 ≥ 10). Note that in the precentral gyrus, there appears a significant difference between severe and non-severe depressed groups.

Finally, to check for potential differences between the non-severe (N = 14) and severe (N = 6) depression groups, we exported the values from each of the significant clusters identified in group and regression analyses in the 0.01–0.08 Hz range (DLPFC, precentral gyrus, and insula). Using the Mann-Whitney-Wilcoxon Test to compare non-severe and severe groups, we found no difference in the DLPFC (p = 0.30), no difference in the insula (p = 1), but a significant difference in the precentral gyrus (p = 0.043) between the groups, suggesting that the effect in the precentral gyrus was driven by the severely depressed participants (see Fig. 4 showing the result for the non-severe and severe groups in comparison with non-depressed participants).

## 4 Discussion

In this study, we used resting state fALFF to identify brain correlates of post-stroke depression. Despite being a major approach to studying post-stroke depression, the analysis of stroke lesions, both lesion volume and location, did not reveal any significant group differences or association with depression severity. By contrast, with fALFF in the 0.01–0.08 Hz range, we observed a significant difference between depressed and non-depressed stroke patients in the left DLPFC and the right precentral gyrus, with both regions showing higher fALFF in the depressed group. Depression severity assessed with PHQ-9 was associated with higher fALFF in the left insula. When we repeated the analysis for the slow-5 and slow-4 ranges separately, we found that the higher right precentral gyrus fALFF observed in the group comparison was mostly contributed by the slow-5 oscillations. The left insula correlation with depression severity was mostly attributable to the slow-4 oscillations. Together these findings suggest that the typical range fALFF is associated with post-stroke depression with distinct contributions from slow-5 and slow-4.

Our main goal was to identify brain regions where local neural activity expressed through the amplitude of low frequency fluctuations was associated with depression symptoms. Consistent with prior studies of depression in general and post-stroke depression in particular, we observed aberrant spontaneous neural activity in the insular cortex (Cohen et al., 2013, Fang et al., 2017, Kuhn and Gallinat, 2013, Li et al., 2014, Ma et al., 2013, Soriano-Mas et al., 2011, Sprengelmeyer et al., 2011, Veer et al., 2010, Wiebking et al., 2011) and the left DLPFC (Downhill and Robinson, 1994, Fales et al., 2008, Grajny et al., 2016, Hwang et al., 2015, Liu et al., 2014, Ma et al., 2013, Sheline et al., 2010, Vasic et al., 2009). In addition, we observed higher fALFF in the right precentral gyrus that has not been strongly linked to depression but nevertheless reported in several previous studies, mostly showing reduced gray matter volume in this region in both major and subthreshold depression (Grieve et al., 2013, Taki et al., 2005). The insula, with its extensive connections to fronto-limbic areas as part of the saliency network, has previously been linked to aberrant emotional, and interoceptive processing in depression (Sliz and Hayley, 2012). The DLPFC and precentral gyrus, as part of the cognitive control network, have been implicated in the cognitive impairment associated with depression (Williams, 2016). Of interest is that further analysis of the non-severe and severe depression groups in our study revealed that increased fALFF in the DLPFC and the insula reflected the presence of depressive features, while an increase in fALFF in the precentral gyrus was related to the severity of depression in that only the more severely depressed participants showed increased fALFF in this region.

While the loci showing aberrant fALFF are consistent with previous literature, the direction of the effects requires further discussion, which is complicated by the lack of a clear understanding of the relationship between fALFF and other measures of brain functioning necessary to relate the current findings to existing literature. Several studies have shown a positive correlation between low frequency fluctuations and resting state PET measuring fluorodeoxyglucose metabolism (Aiello et al., 2015), functional connectivity (Di et al., 2013, Zhang et al., 2014) and functional activation (Zhang et al., 2014), and negative correlation with cortical thickness (Zhang et al., 2014). In line with this, we observed higher fALFF in the right precentral gyrus, consistent with the higher fALFF previously reported in this region (Wang et al., 2012) in depressed participants, and with the decreased gray matter volume in this region (Grieve et al., 2013, Taki et al., 2005). The findings of the higher fALFF in the left DLPFC and left insula, however, are only partially consistent with previous literature. Both showed higher fALFF in our study, consistent with the previous findings of lower gray matter volume in depressed participants in the left DLPFC (Grieve et al., 2013) and insula (Cohen et al., 2013, Hatton et al., 2012, Soriano-Mas et al., 2011). Several studies, however, reported hypo-activation and lower connectivity of the left DLPFC (Kaiser et al., 2015, Wolkenstein and Plewnia, 2013), including a fALFF study that showed lower fALFF in the DLPFC in depressed participants (Wang et al., 2012). Similarly, there are many reports of insula hypo-activation in functional tasks (Fitzgerald et al., 2008). Assuming that fALFF positively correlates with activation levels and connectivity, higher fALFF in DLPFC and insula observed here would be inconsistent with the hypothesised direction of the effect. At the same time, there is no full agreement on whether, for instance, insula is hyper-or hypo-activated, with variable reports depending on the use of positive or negative stimuli, the stage (first-episode vs. repeated) or severity (major vs. subthreshold) of depression (Sliz and Hayley, 2012), or even whether depressed patients are more likely to respond to drug or cognitive behaviour therapy (McGrath et al., 2013). Equally, there is no consensus on the relationship between the different brain imaging measures, and no normative level of fALFF. Further studies are needed to elucidate the functional significance of spontaneous low frequency fluctuations in depression. What is clear is that given the overlap of the regions showing vulnerability in depression measured with different methods, including fALFF reported here, structural and functional, task-based and resting state, connectivity and local activity measures should be investigated in concert to reveal functional links between them.

An advantage of measuring fALFF compared to other resting state measures is that it allows the analysis of frequency-specific activity. The areas revealed in these analyses - DLPFC/precentral gyrus vs. the insula in the slow-5 and slow-4 ranges respectively - were consistent with the sensitivity within these frequency bands. Slow-5 oscillations are known to better reflect signal from the cortical regions, whereas slow-4 fALFF is more sensitive to subcortical regions (Wang et al., 2016), including the insula (Kalcher et al., 2014). In addition lower frequencies have been linked to long-range connectivity, involving the brain's integration hubs, while higher frequencies were related to more local neural activity and shorter connections (Wang et al., 2016). Our frequency-specific results are consistent with these intrinsic differences between slow-4 and slow-5. While the physiological sources of the different bands are somewhat known (Wang et al., 2016), little is known about the pathological implications. This study is one of the first to report post-stroke depression-related differences in sub-bands and necessitates further research. Group differences in the current study were mostly observed in slow-5 band and correlations in slow-4 oscillations. One possibility is that there could be a systematic difference between what these analyses detect in different bands of low frequency fluctuations. Previous fALFF studies in stroke and depression suggested that the slow-5 could be more sensitive for detection of depression after stroke (La et al., 2016, La et al., 2016, Wang et al., 2016). In these previous studies a group contrast was used. Here, we also report more sensitivity of slow-5 to the contrast between depressed and non-depressed patients.

The brain regions revealed by the group analysis in the full range 0.01–0.08 Hz included the right precentral gyrus, lateral to the right frontal eye fields (FEF) area implicated in dorsal attention (He et al., 2007), and the left DLPFC, typically associated with the cognitive control network (Cole and Schneider, 2007). It is therefore possible that the group differences here reflect specific comorbidities associated with depression. For example, cognitive impairments are both associated with - and co-vary with - depression. Cognitive impairment in post-stroke depression has been specifically related to the left DLPFC (Downhill and Robinson, 1994). By contrast, a regression with the PHQ-9 scores revealed the relevance of the left insula in both 0.01–0.08 Hz and slow-4. The insula is critically associated with interoceptive and emotional salience processing (Avery et al., 2014, Menon and Uddin, 2010). Patients with depression are known to exhibit abnormalities with interoception (Harshaw, 2016, Khalsa and Lapidus, 2016). Therefore, specificity of fALFF sub-bands in post-stroke depression should be further investigated to unveil their contributions to brain functioning and behaviour.

### 4.1 Limitations and future directions

The current study directly compared stroke participants with and without depression, focussing on the influence of depression on the brain after stroke. An important question to address in future studies in stroke and healthy populations would be an interaction between depression and stroke, disentangling the effect of the presence of stroke and presence of depression.

We studied depression cross-sectionally, at 3 months post-stroke. This, however, limits our ability to conclude whether stroke event has a causal connection to the fALFF changes observed. Future longitudinal studies are needed to identify progression of disease and respective changes in low frequency oscillations. Likewise, the fALFF measure does not provide information about causality. It does, however, reveal certain patterns in brain activity that can be further investigated for therapeutic purposes, e.g., the left-right hemisphere asymmetry in slow oscillations can be estimated with the prospect of applying neurofeedback or non-invasive neuromodulation to restore the balance.

## 5 Conclusion

We found that post-stroke depression was associated with higher fALFF in the left DLPFC and the right precentral gyrus, with depression severity in all patients correlated with the left insula fALFF. Group differences were revealed in slow-5 fluctuations and depression severity correlates were found in the slow-4 range, possibly reflecting different aspects of the depressive disorder. These fALFF results complement and enrich our understanding of resting state functioning in depression. Compared to the lesion-symptom mapping, the fALFF measure appears to be sensitive even in a smaller sample with diverse lesion distribution.

## Conflict of interest declaration

Authors report no conflict of interest.
